# Effect of Persian and almond gums as fat replacers on the physicochemical, rheological, and microstructural attributes of low‐fat Iranian White cheese

**DOI:** 10.1002/fsn3.446

**Published:** 2016-12-02

**Authors:** Hossein Jooyandeh, Mostafa Goudarzi, Hadis Rostamabadi, Mohammad Hojjati

**Affiliations:** ^1^ Department of Food Science and TechnologyRamin Agricultural and Natural Resources UniversityAhvazIran; ^2^ Department of Food Science, Technology and EngineeringUniversity College of Agriculture and Natural ResourcesUniversity of TehranKarajIran

**Keywords:** Low‐fat Iranian White cheese, rheology, persian gum, almond gum, RSM

## Abstract

The effect of Persian and almond gums (0, 0.1 and 0.2% (w/w)) as fat replacers and milk fat (0.4, 0.9, and 1.4% (w/w)) on physicochemical and rheological characteristics and microstructure of low‐fat Iranian White cheese was studied. Persian and almond gums both effectively increased moisture‐to‐protein (M:P) ratio of low‐fat cheese samples which in turn led to a significant reduction in the hardness parameters fracture stress and Young's and storage (G’) moduli (*p* < .05); however, the effect of Persian gum was more pronounced (*p* < .01). Gum addition promoted cheese yield and proteolysis rate (*p* < .05). Response surface optimization described that supplementation of cheese milk containing 0.9% fat with 0.2% Persian gum and 0.12% almond gum would result in a low‐fat cheese with textural properties similar to its full‐fat counterpart. Scanning electron microscopy revealed that the fat replacers produced full‐fat‐like structure in the low‐fat Iranian White cheese, when incorporated at the optimum levels.

## 1 Introduction

Growing awareness of the role of obesity in development of diseases such as diabetes mellitus type 2, coronary heart disease, and certain type of cancers had led to an explosion of research into developing low‐fat versions of food products (Sayadi, Madadlou, & Khosroshahi, 2013). Development of low‐fat foods that look and taste like their full‐fat counterparts has always been a challenge for food technologists. Low‐fat cheeses usually suffer from over‐firm and elastic texture as well as lower yield and slower ripening due to protein‐dominated structure of such products (Diamantino, Beraldo, Sunakozawa, & Penna, 2014). Employing hydrocolloids as fat replacer in formulation of low‐fat cheeses is an attractive strategy to combat problems associated with fat reduction. They improve the properties of low‐fat cheeses by binding extra water which creates the functional and organoleptic properties similar to fat (Kavas, Oysun, Kinik, & Uysal, 2004). A variety of hydrocolloids have been used in the formulation of low‐fat cheeses to simulate the functional and organoleptic properties of fat, including starch, xanthan, carrageenan, guar, pectin, alginate, locust bean gum, gelatin, inulin, gum Arabic, and basil seed gum (Hosseini‐Parvar, Matia‐Merino, & Golding, 2014).

Persian gum is a novel non‐starch water‐soluble exudate gum from the wild or mountain almond trees (*Amygdalus scoparia* Spach) (Ghasempour, Alizadeh, & Bari, 2012). Persian gum is transparent to semicloudy with no special flavor and its properties are similar to gum Arabic (Abbasi & Mohammadi, 2013). Ghasempour et al. (2012) showed that Persian gum as a fat replacer improved the viscosity of low‐fat set yogurt and stabilized the yogurt gel against syneresis during storage. They attributed the stabilizing effect of Persian gum on yogurt gel to its interactions with milk proteins. The preventing effect of Persian gum on phase separation in dairy products has also been reported for Doogh, a yogurt‐based Iranian drink (Nabizadeh, Khosrowshahi, & Zomorodi, 2014). Almond gum is another novel gum exuded from the trunk, branches, and fruits of *Prunus dulcis* trees, after mechanical damage or infection by microorganisms (Mahfoudhi et al., 2014). Almond gum is mainly composed of polysaccharides (92.36%) including arabinose (46.83%), galactose (35.49%), and uronic acid (5.97%), with a small amount of protein (2.45%) (Mahfoudhi, Chouaibi, Donsì, Ferrari, & Hamdi, 2012). Almond gum has been shown to exert stabilizing effect on oil‐in‐water emulsions comparable to gum Arabic (Mahfoudhi et al., 2014). It is believed that protein fractions of almond gum as well as its arabino‐galactan fraction are main factors responsible for its interfacial activity (Mahfoudhi et al., 2014). However, potential application of theses gums as fat replacer has not so far been explored in the formulation of low‐fat cheeses.

Iranian White cheese is a close textured brined cheese consumed throughout the country as the main breakfast item (Rahimi, Khosrowshahi, Madadlou, & Aziznia, 2007). It is of interest to explore potential application of almond gum and Persian gum as fat replacer for development of low‐fat Iranian White cheese. Determination of the optimum levels of fat replacers for development a low‐fat cheese with matching quality of its full‐fat counterpart is a cost‐ and time‐consuming process which is usually carried out by trial and error until finding the optimum formulation. Response surface methodology (RSM) is a beneficial tool for optimizing the formulation of new products. It minimizes the number of experimental trials required and defines mathematical models to evaluate the influence of independent variables on the responses of interest (Goudarzi, Madadlou, Mousavi, & Emam‐Djomeh, 2015). Recently, RSM was successfully employed by Lashkari, Khosrowshahi, Madadlou, and Alizadeh (2014) to optimize the rheological properties of low‐fat Iranian White cheese supplemented with fat replacers. The main objective of this study was to optimize the formulation ingredient of low‐fat Iranian White cheese including almond gum and Persian gum as fat replacers and fat content in order to develop a low‐fat cheese with textural attributes similar to its full‐fat counterpart using response surface method.

## 2 Materials and Methods

### 2.1 Materials

Raw skim milk and cream were supplied by Khuzestan Pegah Dairy Company (Shush, Iran). Two freeze‐dried direct‐to‐vat mixed starter cultures (CHOOZIT 230 and YO‐MIX 532) were obtained from Danisco Deutschland GmbH (Alemanha, Germany). Culture CHOOZIT 230 contained *Lactococcus lactis* subsp. *cremoris* and *L. lactis* subsp. *lactis*. Culture YO‐MIX 532 contained *Streptococcus thermophilus* and *Lactobacillus delbrueckii* subsp. *bulgaricus*. Standard cheese‐making rennet (2235 international milk clotting units g^−1^) was procured from Chr. Hansen's Laboratory Ltd. (Hørsholm, Denmark). Almond gum and Persian gum as fat replacers were collected directly from the trees of mountainous regions in Shiraz and Khouzestan provinces (Iran), respectively. The gums were powdered in a high‐speed mechanical blender and then sieved to obtain a uniform particle size. The powders were used without any further purification. All other solvents and reagents used were of analytical grades and obtained from Merck Chemicals Ltd. (Darmstadt, Germany).

### 2.2 Cheese‐making procedure

Raw skim milk was standardized with cream to achieve three target fat contents of 0.4, 0.9, and 1.4%. For each batch of cheese, 4 kg standardized milk was heated to 35°C, supplemented with different levels of Persian and Almond gums (0, 0.1, and 0.2% w/w) and then batch‐pasteurized at 64°C for 30. After pasteurization, batches of milk were cooled to 35°C and supplemented with CaCl_2_ at a rate 0.1 g per kg of milk. This was followed by inculcation of cheese milk with 0.04 g L^−1^ starter culture. The milk was held at 35°C for 55 min for starter maturation before adding the rennet (0.025 g L^−1^). The curd was cut into approximately 1‐cm cubes 45 min after addition of rennet and allowed to remain quiescent for 10 min while whey expulsion was initiated. The cut curds were then gently agitated for 10 min to facilitate whey expulsion and subsequently pressed for 2.5 h to complete the draining. The pressed curd was cut into pieces of 4 × 6 × 6 cm, packed in airtight plastic bags, covered with pasteurized brine (13% salt concentration), and kept refrigerated for a ripening period of 60 days (Sayadi et al., 2013). The control full‐fat cheese (milk fat: 3%) and control low‐fat cheese (milk fat: the same as optimized sample) were produced in a similar method, but without adding the gums.

### 2.3 Experimental design and statistical analysis

RSM was employed to optimize the formulation ingredients of low‐fat Iranian White cheese according to a 3‐level‐3‐factor Box–Behnken design. The experimental design with coded and actual values is presented in Table 1. The design consisted of 15 experiments with three center points to calculate the repeatability of the method. According to experimental design, cheese samples were prepared with varied concentration of milk fat, Persian gum, and almond gum and evaluated for physicochemical and rheological characteristics at the day 60 of the ripening period. A second‐order polynomial equation was fitted to the obtained experimental data for responses:

(1) $$y=\beta_{0}+\sum_{i=1}^{3}\beta_{i}X_{i}+\sum_{i=1}^{3}\beta_{\mathrm{ii}}X_{i}^{2}+\sum_{i=1}^{2}\sum_{j=i+1}^{3}+\beta_{\mathrm{ij}}X_{i}X_{j}+\varepsilon_{\mathrm{ij}}$$

**Table 1:** The RSM experimental design for development of low‐fat Iranian White cheese incorporated with Persian and almond gums as fat replacers

Standard order	Independent variables					
	Coded levels			Uncoded levels		
	X1	X2	X3	Persian gum (% w/w)	Almond gum (% w/w)	Milk fat (% w/w)
1	0	−1	−1	0.1	0.0	0.4
2	+1	0	−1	0.2	0.1	0.4
3	−1	0	−1	0.0	0.1	0.4
4	0	−1	+1	0.1	0.0	1.4
5	0	+1	−1	0.1	0.2	0.4
6	0	0	0	0.1	0.1	0.9
7	0	0	0	0.1	0.1	0.9
8	+1	−1	0	0.2	0.0	0.9
9	0	0	0	0.1	0.1	0.9
10	−1	+1	0	0.0	0.2	0.9
11	0	+1	+1	0.1	0.2	1.4
12	+1	+1	0	0.2	0.2	0.9
13	−1	−1	0	0.0	0.0	0.9
14	+1	0	+1	0.2	0.1	1.4
15	−1	0	+1	0.0	0.1	1.4

where Y is the response (physicochemical and rheological characteristics), β_0_, β_i_, β_ii_, and β_ij_ are regression coefficients for intercept, linear, quadratic, and interaction coefficients, respectively, and X_i_ and X_j_ are the independent variables (Persian gum, almond gum, and milk fat). Response surface analysis, mapping of plots, and response optimization were performed using statistical package Design‐Expert, version 7.0.0 (Stat‐Ease Inc., Minneapolis).

### 2.4 Physicochemical analysis

Samples were analyzed for moisture content by vacuum‐oven method (AOAC, 1997). To determine protein content, total nitrogen content of the cheese samples was measured by Kjeldahl method (AOAC, 1997) and a factor of 6.38 was used to convert nitrogen content to protein content. Water‐soluble nitrogen (WSN) was determined according to a method described by Karami, Ehsani, Mousavi, Rezaei, and Safari (2009a) and the results were expressed as percentage of total nitrogen in the cheese. The cheese yield was calculated as kilograms of cheese (before binning) per 100 kg of milk used (Sayadi et al., 2013). The analyses were performed in triplicate.

### 2.5 Rheological analysis

#### 2.5.1 Uniaxial compression

Cheese samples were cut into cubes of 1.5 cm and equilibrated at room temperature (20 ± 1°C) in airtight plastic containers for 4 hr before the compression testing. The cheese samples were compressed to 80% of their original size at the speed of 30 mm min^−1^ using a texture analyzer (TA‐XT2i texturometer, Stable Micro Systems, England) equipped with an aluminum cylindrical probe of 36 mm diameter (Juan, Zamora, Quintana, Guamis, & Trujillo, 2013). The stress required to fracture the sample was calculated using following equation:

(2) σ=F/A

where σ (kPa) is the fracture stress, F (N) is the force recorded in each test, and A (cm^2^) is initial cross‐sectional area of each sample. The secant modulus at the fracture point was measured as the elastic modulus of the cheeses (Sayadi et al., 2013).

#### 2.5.2 Dynamic oscillatory measurements

Small‐amplitude oscillatory shear measurements were performed using a Paar Physica universal dynamic spectrometer (UDS 200, Physica Messtechnik GmbH, Stuttgart, Germany) with 25‐mm diameter parallel plates. Cheeses were sliced to a 1‐mm thickness and placed on the lower plate of measuring geometery; the upper plate was then lowered until it reached a 1‐mm gap distance and the sample was trimmed. The sample was allowed to relax for 20 min prior to oscillation. A frequency sweep test over a range of 0.01–100 Hz was performed at constant strain amplitude of 0.5%. The storage modulus (G’) was determined.

#### 2.5.3 Microstructure

Scanning electron microscopy (SEM) was performed on cheese samples at day 60 of ripening, following the methodology described by Sayadi et al. (2013). Cheese samples were cut into cubes of approximately 5 mm^3^ and fixed through immersion in 2.5% (w/w) gluteraldehyde for 3 hr. After fixation, the cheese blocks were washed in distilled water and dehydrated using a graded (40, 55, 70, 85, 90, and 96%) series of ethanol for 30 min each. The samples were then defatted three times in chloroform for 10 min each. The defatted samples were covered with ethanol and kept refrigerated until they were freeze‐fractured in liquid nitrogen into approximately 1‐mm pieces. The samples were mounted on aluminum SEM stubs with silver paint, dried to critical point, and coated with gold for 6 min in a sputter‐coater (Balzers, type A450x; Baltek Inc., Pfäffikon, Switzerland). The scanning electron micrographs were taken with a scanning electron microscope (TESCAN, model VEGA; Brno, Czech Republic) operated at 15.0 kV. Photomicrographs were recorded at 500, 1000, 2000, and 5000 magnification levels. Using an image analysis software (Image J, National Institutes of Health, Bethesda, Maryland), two‐dimensional images of SEM were changed to three‐dimensional (3D) images (Karami, Ehsani, Mousavi, Rezaei, & Safari, 2009b).

## 3 Results and Discussion

### 3.1 Physicochemical characteristics

**Figure 1:**
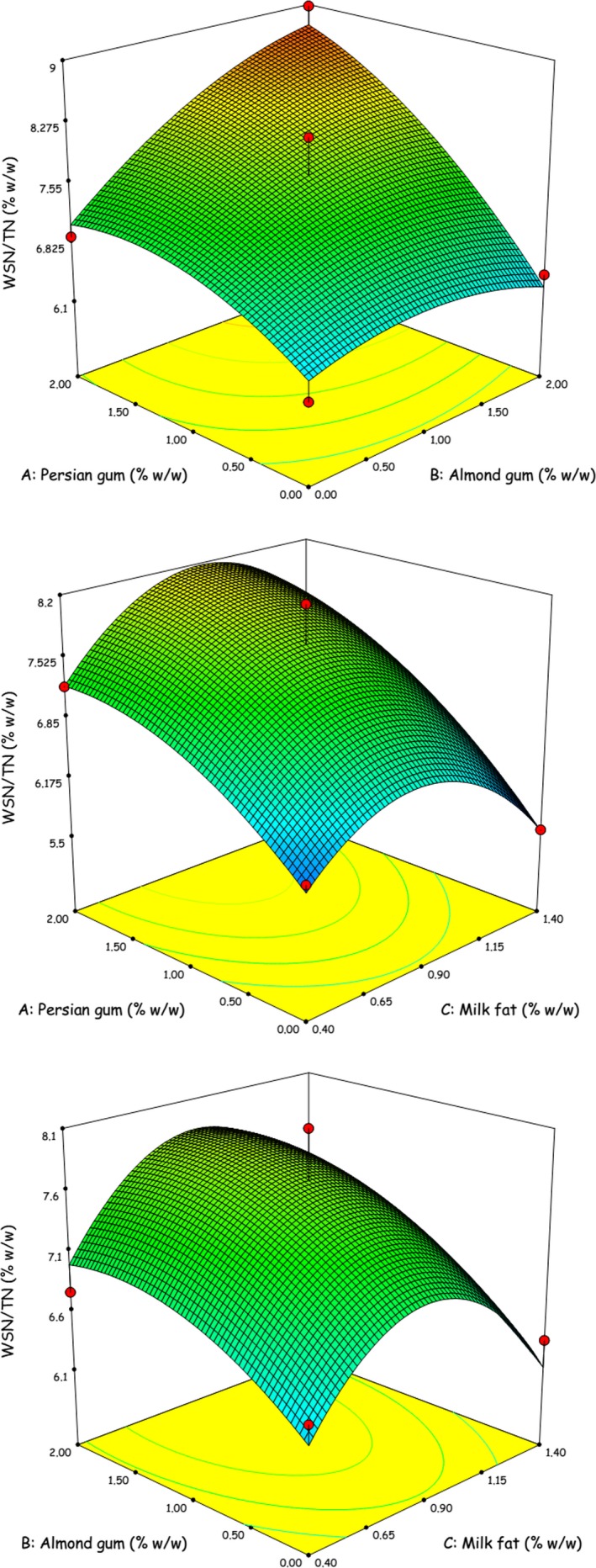
Response surface plots for interaction effects of formulation ingredients on WSN/TN content of low‐fat Iranian White cheese incorporated with Persian and almond gums as fat replacers

The results of statistical analysis for the effects of fat reduction and gum addition on the physicochemical attributes of 60‐day‐old Iranian White cheese are shown in Table 2. It was observed that fat reduction was accompanied by significant increase in moisture and protein contents of the cheese samples. However, their moisture to protein (M:P) ratio experienced a significant reduction as the fat content decreased. This implies that the moisture did not replace the fat on an equal basis in the low‐fat cheese samples. Similar results have been reported by other researchers (Cooke, Khosroshahi, & McSweeney, 2013; Rahimi et al., 2007; Romeih, Michaelidou, Biliaderis, & Zerfiridis, 2002). Persian and almond gums owing to their water‐holding properties both caused a considerable increase in moisture content of the low‐fat cheeses which in turn led to lower protein content and higher M:P ratio for these samples (Table 2). Increased M:P ratio in gum‐containing cheese samples promoted the WSN/TN ratio as the index of ripening extension (Table 2). This can be attributed to higher rate of proteolysis occurring in cheeses with higher moisture in non‐fat substance (MNFS) due to greater accessibility of proteolytic enzymes to protein substrate (Zisu & Shah, 2005). Rahimi et al. (2007) observed more extensive proteolysis in low‐fat Iranian White cheese incorporated with gum tragacanth as fat replacer, when compared to low‐fat control cheese at various stages during ripening. As shown by the response surface graphs (Figure 1), production of WSN progressively increased by decreasing the fat content until a certain level but further decrease in fat content contributed in reduction of WSN production. It is generally assumed that higher amounts of soluble chymosin in low‐fat cheeses with greater moisture contents are responsible for more proteolysis and hereby more production of WSN (Rudan, Barbano, Yun, & Kindstedt, 1999). It seems, however, that at higher levels of fat reduction, decreasing the proteolysis rate due to insufficient M:P ratio is more likely to occur (Rudan et al., 1999). Decreasing effect of fat reduction on proteolysis rate has previously been reported for Mozzarella (Rudan et al., 1999), Cheddar (Cooke et al., 2013) and Minas cheeses (Diamantino et al., 2014). The results indicated that cheese yield tended to decrease significantly as the fat content was reduced (Table 2). Since the moisture increase was not great enough to offset the decrease in the fat content as indicated by decreased M:P ratio, the total filler volume (fat and moisture) of casein matrix in the low‐fat cheeses underwent a reduction which consequently led to a lower cheese yield (Rudan et al., 1999). Incorporation of Persian gum or almond gum within the cheese curd markedly increased the yield (Table 2). Improved yield is because of water‐keeping ability of the gums which increased retention of serum in the cheese matrix (Rahimi et al., 2007). Our findings on the effects of fat reduction and fat replacer addition on cheese yield are in agreement with the literature (Diamantino et al., 2014; Kavas et al., 2004; Rahimi et al., 2007; Romeih et al., 2002).

**Table 2:** Analysis of variance (ANOVA) of the physicochemical characteristics of the low‐fat Iranian White cheese incorporated with Persian and almond gums as fat replacers

Source	Moisture		Protein		M:P		WSN/TN		Yield	
	Coefficient	*p*‐value	Coefficient	*p*‐value	Coefficient	*p*‐value	Coefficient	*p*‐value	Coefficient	*p*‐value
Intercept	69.926	.000	25.416	.000	2.752	.000	7.716	.000	13.140	.000
X_1_	1.998	.004	−1.227	.001	0.239	.000	0.790	.001	0.702	.003
X_2_	1.035	.044	−0.561	.026	0.117	.010	0.403	.019	0.327	.049
X_3_	−5.706	.000	−4.106	.000	0.285	.000	−0.033	.786	1.822	.000
X_1_. X_1_	−0.428	.487	0.160	.570	−0.018	.689	−0.334	.112	−0.152	.469
X_2_. X_2_	−0.335	.582	0.032	.906	−0.020	.647	−0.277	.171	−0.312	.170
X_3_. X_3_	−1.853	.023	−2.207	.000	0.232	.003	−0.827	.005	−0.412	.088
X_1_. X_2_	−0.177	.759	0.275	.328	−0.027	.527	0.440	.046	0.055	.781
X_1_. X_3_	0.705	.255	−0.445	.140	0.150	.014	0.170	.355	0.060	.762
X_2_. X_3_	0.742	.234	−0.262	.348	0.098	.060	0.057	.744	0.095	.634
Regression	—	.001	—	.000	—	.001	—	.10	—	.001
Linear	—	.000	—	.000	—	.000	—	.004	—	.000
Quadratic	—	.100	—	.002	—	.014	—	.019	—	.197
Interaction	—	.401	—	.268	—	.034	—	.156	—	.926
Lack of fit	—	.432	—	.927	—	.242	—	.533	—	.114
*R* ^2^	.981		.992		.979		.948		.979	
*R* ^2^‐adjust	.948		.978		.941		.854		.941	

X_1_, X_2_, & X_3_ are Persian gum, almond gum, and milk fat, respectively.

### 3.2 Rheological characteristics

The reduction in fat content of Iranian White cheese significantly adversely influenced the cheese texture in terms of rheological parameters including fracture stress and Young's and storage (G’) moduli (Table 3). Madadlou, Khosroshahi, and Mousavi (2005) found that the low‐fat cheese samples possessed significantly higher stress at fracture and Young's modulus of elasticity compared to the control full‐fat cheese. The fracture stress and Young's modulus are associated with cheese softness; that is, the higher the uniaxial compression parameters, the greater the cheese firmness (Rahimi et al., 2007). In agreement with our date, Zalazar et al. (2002) showed that fat reduction of cheese was concomitant with increased rheological parameters, especially parameter G’, indicating a more elastic texture. Fat acts as plasticizer between the casein chains, providing the typical softness of a full‐fat cheese (Karami et al., 2009a). Hence, an absence of fat allows more protein contact points in the cheese matrix and thereby, promotes the possibilities of cross‐linking between protein chains leading to a more compact casein matrix and a cheese with hard and rubbery texture (Diamantino et al., 2014). Both Persian and almond gums effectively reduced the rheological parameters of low‐fat Iranian White cheese. However, the effect of Persian gum was more pronounced (Table 3). Rahimi et al. (2007) and Lashkari et al. (2014) reported improved texture with less elasticity and fracture stress for low‐fat Iranian White cheese containing gum tragacanth and a mixture of guar gum and gum Arabic as fat replacers, respectively. It is believed that gums could mimic the physical properties of fat in low‐fat cheeses via binding extra water which interrupts the extensive protein network formed in the absence of fat, resulting in a smoother protein matrix and a less rubbery cheese (Cooke et al., 2013). Likewise, promoted proteolytic breakdown of protein matrix in gum‐containing low‐fat cheese samples (Table 2) could somewhat account for decreased cheese hardness in relation to rheological parameters (Rahimi et al., 2007).

**Table 3:** Analysis of variance (ANOVA) of the rheological attributes of the low‐fat Iranian White cheese incorporated with Persian and almond gums as fat replacers

Source	Fracture stress (σ_f_)		Young's modulus (E)		Storage modulus (G’)	
	Coefficient	*p*‐value	Coefficient	*p*‐value	Coefficient	*p*‐value
Intercept	66.880	.001	666.38	.000	652.667	.000
X_1_	−22.009	.017	−90.72	.005	−121.500	.003
X_2_	−21.042	.020	−50.99	.044	−77.500	.018
X_3_	−60.765	.000	−244.02	.000	−240.500	.000
X_1_. X_1_	0.456	.962	−25.49	.404	−63.833	.112
X_2_. X_2_	−1.533	.874	−39.62	.216	−57.333	.144
X_3_. X_3_	39.609	.008	−24.15	.427	194.667	.002
X_1_. X_2_	−4.148	.658	−69.51	.049	−89.000	.038
X_1_. X_3_	20.759	.065	38.97	.207	64.500	.098
X_2_. X_3_	22.918	.048	18.99	.511	5.500	.869
Regression	—	.003	—	.001	—	.001
Linear	—	.001	—	.000	—	.000
Quadratic	—	.038	—	.448	—	.006
Interaction	—	.079	—	.128	—	.085
Lack of fit	—	.067	—	.170	—	.153
*R* ^2^	.967		.976		.977	
*R* ^2^‐adjust	.909		.934		.936	

X_1_, X_2_, & X_3_ are Persian gum, almond gum, and milk fat, respectively.

### 3.3 Formulation optimization

The optimization of low‐fat Iranian White cheese formulation was conducted with the aim of minimizing the hardness parameters G′, E, and σ_f_. RSM described that supplementation of low‐fat cheese milk (0.9% w/w fat) with 0.2% w/w Persian gum and 0.12% w/w almond gum would provide the softest texture in terms of studied rheological parameters (Table 4). For the model verification in predicting the optimum formulation, cheese sample with the proposed formulation was prepared and its physicochemical and textural characteristics were compared with those predicted by models. The result of one‐sample *t*‐test showed that there were no statistically significant differences between the experimental and estimated values of all responses within a 95% confidence interval except for those of WSN/TN (Table 4); thereby, the adequacy of the models in predicting the optimum formulation was confirmed.

**Table 4:** Physicochemical and Rheological attributes of control full‐fat, control low‐fat, and optimized low‐fat Iranian White cheese

Sample		Physicochemical attributes					Rheological attributes		
		Moisture (% w/w)	Protein (% w/w)	M:P	Yield (%)	WSN/TN (% w/w)	σ_f (_kPa_)_	E (kP)	G’ (kPa)
Optimized low‐fat	Predicted	64.92	17.49	3.67	15.23	9.87	44.45	300.81	454.21
	Experimental	63.07 ± 0.76^a^	16.61 ± 0.55^c^	3.80 ± 0.17^a^	14.55 ± 0.29^b^	7.40 ± 0.12^a^*	48.98 ± 1.91^b^	362.48 ± 25.48^b^	428 ± 11.75^b^
Control low‐fat		53.39 ± 0.70^b^	22.97 ± 0.35^a^	2.32 ± 0.03^c^	11.78 ± 0.11^c^	6.32 ± 0.23^c^	92.45 ± 2.55^a^	628.82 ± 16.47^a^	683.00 ± 16.42^a^
Control full‐fat		49.98 ± 0.61^c^	18.78 ± 0.21^b^	2.66 ± 0.01^b^	15.98 ± 0.18^a^	6.84 ± 0.19^b^	33.58 ± 3.07^c^	192.00 ± 11.89^c^	345.00 ± 9.75^c^

Data are means ± SD (*n* = 3); Asterisk indicates experimental value significantly differs from predicted value (*p* < .05); Means within the same column with different superscripts statistically differ (*p* < .05).

As presented in Table 4, the optimized sample had significantly lower hardness parameters G′, E, and σ_f_ than the control low‐fat cheese. Further, the rheological parameters’ values of the optimum sample were close to those of the corresponding full‐fat control. This can be attributed to promoted M:P ratio of the optimum sample (Table 4) (Sayadi et al., 2013). It should be noted that enhanced proteolytic reactions in the optimum low‐fat sample evidenced by its higher WSN/TN (Table 4) may have also contributed to its softer texture compared to the low‐fat control (Rahimi et al., 2007). It is worthy of mention that the optimum cheese had considerably greater yield than its low‐fat counterpart (Table 4).

**Figure 2:**
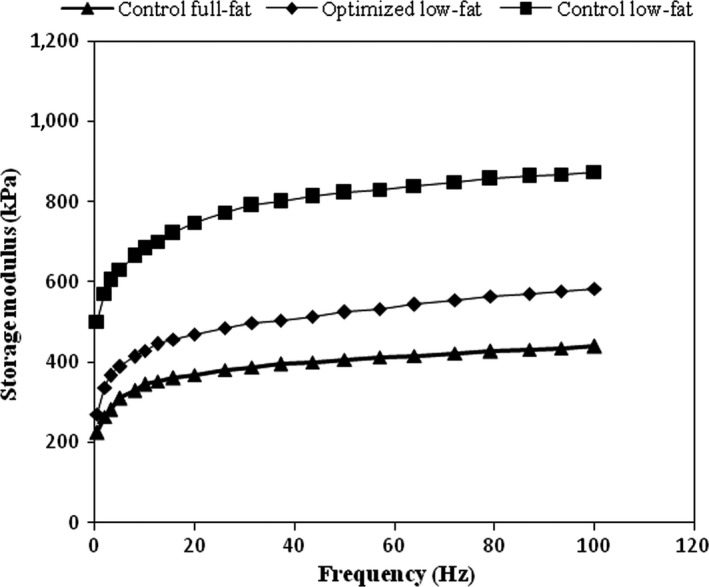
Storage modulus of Iranian White cheese: 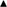, control full‐fat; 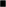, control low‐fat; 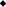, optimized low‐fat

The values of G’ versus oscillation frequency for one replicate of optimized sample and low‐ and full‐fat controls are shown in Figure 2. Storage modulus increased with frequency for all samples reflecting the relaxation of more bonds with more structural rearrangements when the time scale of applied stress was longer (Sayadi et al., 2013). Moreover, all samples had a storage modulus greater than loss modulus (data not shown), indicating their solid‐like behavior. This type of response has previously been reported for Iranian White cheese (Madadlou et al., 2005; Sayadi et al., 2013).

### 3.4 Microstructure

**Figure 3:**
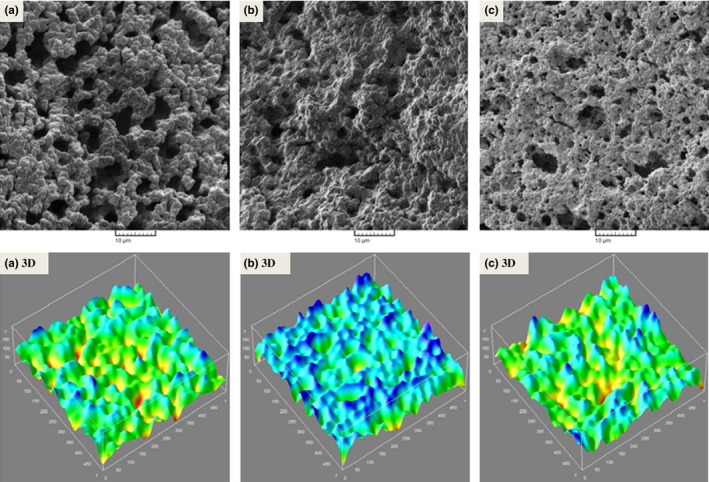
Microstructure of (a) control full‐fat, (b) control low‐fat and (c) optimized low‐fat Iranian White cheese. Tri‐dimensional (3D) images of SEM micrographs are shown below the original images

The scanning electron micrographs of optimized sample and low‐ and full‐fat control cheeses are presented in Figure 3. It can be seen that the low‐fat control sample had the densest protein matrix. The lower the fat content, the fewer are interruption sites in the casein matrix and hence, protein matrix becomes more compact and less voids form (Karami et al., 2009b). Addition of the optimum levels of Persian and almond gums increased the openness of low‐fat cheese structure so that the optimized sample resembled the full‐fat control cheese (Figure 3). This justifies our observation on lower hardness parameters of the optimized low‐fat cheese in comparison with the low‐fat control. Full‐fat‐like microstructure of low‐fat cheese incorporated with fat replacers has been observed by other researchers (Crites, Drake, & Swanson, 1997; Mcmahon, Alleyne, Fife, & Oberg, 1996; Rahimi et al., 2007). Rahimi et al. (2007) speculated that spaces occupied by additional water held by fat replacer in the casein matrix contributed to more open microstructure of low‐fat Iranian White cheese.

## 4 Conclusion

This research provides information on how the physicochemical, rheological, and microstructural properties of low‐fat Iranian White cheese may be altered by adding Persian and almond gums as fat replacers into the formulation. The results showed that the gums increased M:P ratio of low‐fat cheese samples, leading to higher cheese yield and proteolysis rate and lower hardness parameters fracture stress and Young's and storage moduli. It is concluded that by incorporating the optimum levels of almond and Persian gums into the formulation of low‐fat Iranian White cheese, it is possible to develop a low‐fat food with textural properties similar to its full‐fat counterpart.

## Conflict of Interest

None declared.
